# INFLAMMATORY BIOMARKERS ASSOCIATED WITH BRAIN MRI MEASURES: FRAMINGHAM HEART STUDY OFFSPRING COHORT

**DOI:** 10.1093/geroni/igad104.2947

**Published:** 2023-12-21

**Authors:** Jiachen Chen, Margaret Doyle, Yuan Fang, Ahmed A Y Ragab, Joanne Murabito, Kathryn Lunetta

**Affiliations:** Boston University School of Public Health, Boston, Massachusetts, United States; University of Vermont, Burlington, Vermont, United States; Binghamton University, Binghamton, New York, United States; Boston University School of Public Health, Boston, Massachusetts, United States; Boston University Chobanian & Avedisian School of Medicine, Boston, Massachusetts, United States; Boston University School of Public Health, Boston, Massachusetts, United States

## Abstract

Brain MRI volumes measuring brain atrophy have been associated with risks for Alzheimer’s Disease (AD) and related dementia. Inflammatory biomarkers associated with brain MRI volumes may provide insight into the neuroinflammation associated with these diseases. The study aim is to identify circulating inflammatory biomarkers associated with total and regional brain MRI volumes in the Framingham Heart Study Offspring cohort. Participants (N=662, 52% female, mean age 62 years) free of dementia and stroke at the time of blood draw and who had MRI measures within five years were profiled using the OLINK Proteomics inflammation panel. Pairwise cross-sectional associations between 68 biomarkers and eight brain MRI volumes were investigated using linear mixed-effect models accommodating familial correlations and adjusting for covariates (age, age2, sex, time between blood draw and MRI measurement, age-sex interaction, and number of APOE ε2 and ε4 alleles), using FDR≤0.1 to declare significance. APOE genotype-stratified analyses were performed to explore effect modification. Higher levels of 8 proteins were significantly associated with smaller total brain volumes (TCBV), including CDCP1, HGF, IL6, IL8, MMP10, OPG, VEGFA, and 4E-BP1. Higher levels of SCF and TWEAK were significantly associated with larger TCBV, and higher levels of SCF were also associated with larger parietal gray matter volume. In APOE ε4 carriers, higher levels of IFNγ were associated with greater white matter hyperintensity volumes. Consistent with our findings, SCF has been shown to have neuroprotective effects in animal models. Further studies are needed to confirm these potential risk and protective factors and to elucidate mechanisms.

